# Neutrophil-to-lymphocyte ratio is associated with 30-day mortality in acute pulmonary embolism across European Society of Cardiology risk categories

**DOI:** 10.1016/j.rpth.2026.106918

**Published:** 2026-08-21

**Authors:** Umar Hashim, Lizbeth Cabrera, Romil Patel, Mohammad Salah, Yama Sadozai, Kambiz Zorriasateyn, Arman Qamar, Manuel Monreal, Alfonso Tafur

**Affiliations:** 1 Endeavor Health, Vascular Medicine, Skokie, Illinois, USA; 2 Pritzker School of Medicine, University of Chicago, Chicago, Illinois, USA; 3 Faculty of Health Sciences, UCAM—Universidad Católica San Antonio de Murcia, CIBER Enfermedades Respiratorias (CIBERES), Madrid, Spain

**Keywords:** inflammatory biomarkers, prognosis, pulmonary embolism

## Abstract

**Background:**

Pulmonary embolism (PE) remains a major cause of cardiovascular morbidity and mortality. The neutrophil-to-lymphocyte ratio (NLR) is a readily available biomarker reflecting inflammatory stress responses and has been proposed as a predictor of cardiovascular outcomes.

**Objectives:**

This study evaluated the association between elevated NLR and 30-day all-cause mortality in patients with acute PE across European Society of Cardiology (ESC) risk categories.

**Methods:**

A retrospective cohort study of adults with acute PE diagnosed by computed tomography pulmonary angiography was conducted within an integrated health system from January 2019 to January 2025. NLR was calculated from admission complete blood counts and categorized as elevated using a prespecified cutoff (≥7). Patients were stratified by ESC risk category. Multivariable logistic regression was used to assess association between elevated NLR and 30-day mortality, adjusting for age, sex, cancer status, and congestive heart failure.

**Results:**

Among 1525 patients with acute PE, 46.4% were ESC low risk, 51.7% intermediate risk, and 1.9% high risk; 30-day all-cause mortality occurred in 6.6% of patients and was markedly higher among patients with elevated NLR in the ESC low-risk (10.4% vs 1.2%) and intermediate-risk (16.2% vs 3.6%) groups. Elevated NLR was independently associated with increased mortality in ESC low-risk (odds ratio, 5.68; 95% CI, 2.09-15.44) and intermediate-risk patients (odds ratio, 4.60; 95% CI, 2.57-8.24). Addition of NLR to the ESC-based model improved discrimination for 30-day mortality (C-statistic, 0.812-0.853; *P* = .003).

**Conclusion:**

Elevated NLR is independently associated with increased 30-day all-cause mortality in patients with acute pulmonary embolism classified as ESC low or intermediate risk. Consequently, NLR may serve as a simple adjunctive marker for refining PE risk stratification; however, prospective validation is needed.

## Essentials

• Neutrophil-to-lymphocyte ratio (NLR) is a simple blood test that may signal risk in blood clots.
• We studied 1525 adults with pulmonary embolism, a lung blood clot, at 1 hospital system.
• A high NLR (≥7) was linked to higher 30-day death risk, even in lower-risk patients.
• Adding NLR to risk tools may flag patients needing closer monitoring, pending further study.

## Graphical abstract

**Figure:**
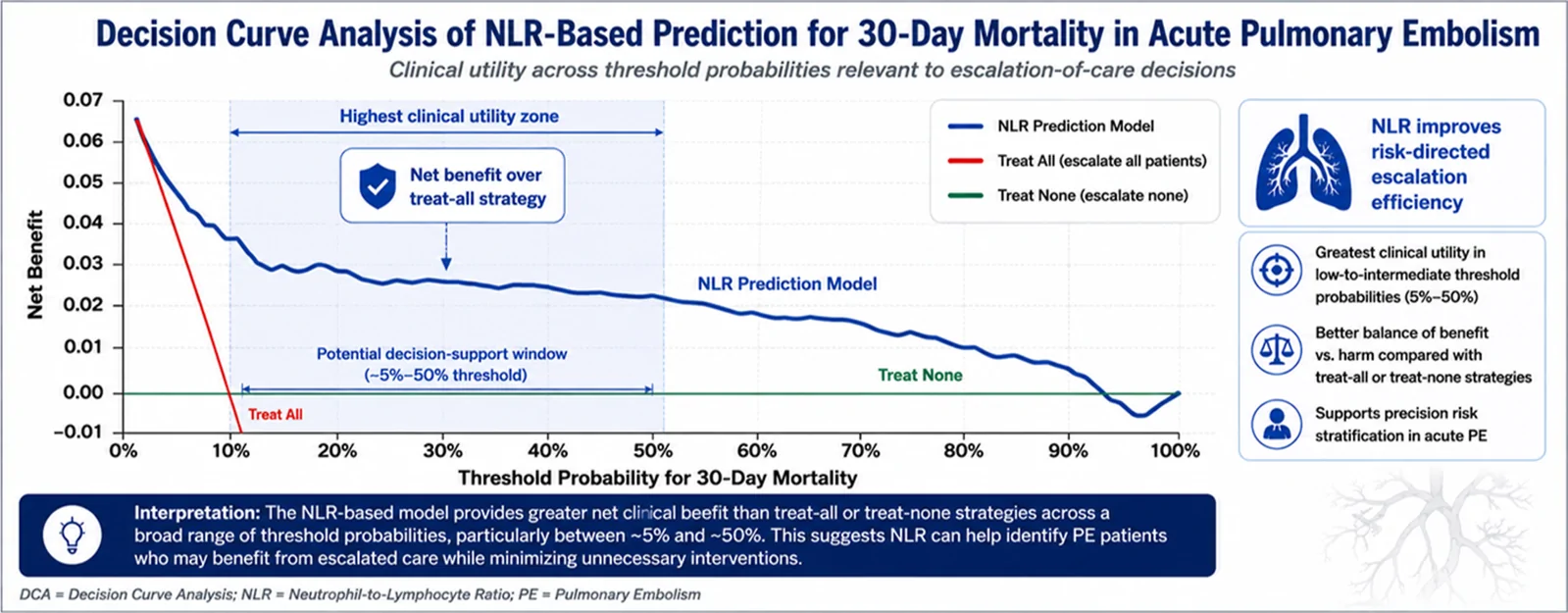

## 1 Introduction

Venous thromboembolism, which encompasses pulmonary embolism (PE) and deep vein thrombosis, remains a major cause of cardiovascular morbidity and mortality, with clinical outcomes ranging from asymptomatic disease to hemodynamic collapse [[1], [2], [3], [4]]. Early risk stratification is central to guiding management decisions, including level of care, monitoring intensity, and consideration of advanced therapies.

Several widely used risk-stratification tools exist, including the Pulmonary Embolism Severity Index (PESI), simplified Pulmonary Embolism Severity Index, and European Society of Cardiology (ESC) classification, which categorizes patients into low-, intermediate-, and high-risk groups. Despite these structured approaches—incorporating clinical features, comorbidities, hemodynamics, right ventricular (RV) dysfunction, and myocardial injury—outcomes remain heterogeneous, particularly among patients classified as low or intermediate risk [5]. Prior studies have shown that the newer ESC algorithms may classify more patients into higher-risk categories without improving prediction of mortality or adverse outcomes, highlighting limitations in current models and suggesting that additional markers capturing aspects of disease severity not reflected in current framework may improve risk stratification [6]. Consistent with this, a subset of these patients—particularly within the intermediate-risk group or those who are normotensive with RV dysfunction under the 2019 ESC framework—experience clinical deterioration or early mortality, indicating that current models may not fully capture underlying biologic risk [[7], [8], [9]]. Emerging evidence suggests a broader shift toward a more individualized approach to risk assessment, including incorporation of biomarkers to complement traditional models [10,11].

Systemic inflammation and physiologic stress are increasingly recognized as contributors to adverse outcomes in vascular disease [12,13]. The neutrophil-to-lymphocyte ratio (NLR), a readily available marker derived from routine complete blood counts, has been associated with adverse outcomes across cardiovascular, pulmonary, renal, and malignant conditions [[14], [15], [16], [17], [18], [19]]. In acute PE, inflammatory activation may contribute to thrombus propagation, endothelial dysfunction, and RV strain, supporting the potential role of NLR as an adjunctive prognostic marker [20,21]. However, the incremental value of NLR within established frameworks such as ESC classification remains unclear, particularly in identifying higher-risk patients within low- and intermediate-risk groups.

In this study, we evaluated the association between elevated NLR and 30-day all-cause mortality in patients with acute PE stratified by ESC risk category. This analysis, conducted in a cohort independent of the RIETE (Registro Informatizado de la Enfermedad TromboEmbólica) registry and Loyola University Medical Center cohort used to derive the prespecified NLR of ≥7 cutoff applied here [22], also provides an independent evaluation, in a cohort separate from the one used to derive the cutoff, of the prognostic association between elevated NLR and 30-day mortality previously reported in that RIETE-based analysis (adjusted odds ratio [OR] 3.46; 95% CI, 2.60-4.60). We also assessed the predictive performance of NLR and its consistency across clinically relevant subgroups, including cancer status, age, and sex.

## 2 Methods

### 2.1 Study design and population

We performed a retrospective cohort study of adult patients (≥18 years) with acute PE diagnosed between January 2019 and January 2025 within an integrated health system. Acute PE was confirmed by computed tomography pulmonary angiography. Patients were stratified according to the European Society of Cardiology (ESC) risk classification (low, intermediate, and high risk) at the time of diagnosis, incorporating hemodynamic status, RV dysfunction, and cardiac biomarkers when available.

### 2.2 Data collection, variables, and definitions

Demographic and clinical data were obtained from the electronic health record including age, sex, a history of congestive heart failure (CHF), and active cancer. Laboratory values at admission were used to calculate the NLR. Chronic kidney disease (CKD) and chronic obstructive pulmonary disease (COPD) status were ascertained by International Classification of Diseases, Tenth Revision, diagnosis code. Admission troponin and brain natriuretic peptide (BNP) status, as incorporated into ESC risk classification, were available for all patients in the analytic cohort. ESC risk category for each patient was assigned from these hemodynamic, biomarker, and imaging data as documented in the electronic medical record at the time of clinical presentation, rather than through independent, blinded post hoc adjudication by the study investigators. NLR was analyzed as a binary variable using a prespecified cutoff of ≥7 (elevated vs nonelevated), consistent with prior studies in acute PE that identified this threshold as optimal for predicting short-term mortality and stratifying patients by risk of adverse outcomes [22]. A data-driven Youden index analysis of this cohort identified an empirically optimal threshold of NLR of ≥6.73 for 30-day mortality; the ≥7 cutoff was retained as the prespecified threshold because it closely approximates this optimum while remaining a simpler, more easily recalled value for clinical use.

The primary outcome was 30-day all-cause mortality, defined from the time of hospitalization. Mortality status was determined through review of the electronic health record, including documented death notifications in the patient chart, communications from family members reporting death, and death certification documentation available within the medical record. Mortality ascertainment was limited to information available within the health system records.

### 2.3 Statistical analysis

Baseline characteristics were summarized using descriptive statistics. Differences between groups were assessed using appropriate univariate methods.

Multivariable logistic regression models were constructed separately within each ESC risk category to evaluate the independent association between elevated NLR (≥7) and 30-day mortality. Covariates were selected a priori based on clinical relevance and included age, sex, active cancer, and CHF. Adjusted associations were reported as odds ratios (ORs) with 95% CIs. To assess robustness of this association, sensitivity analyses sequentially adjusted the multivariable model for CKD, COPD, troponin, BNP status, and thrombocytopenia; tested a formal NLR × cancer status interaction term; and modeled NLR as a continuous variable to assess a dose–response relationship independent of the ≥7 cutoff. Fine–Gray competing risks regression was used to evaluate elevated NLR in relation to PE-specific and non-PE competing death (RIETE fatal PE definition), in the full cohort and separately within the cancer subgroup. Determinants of NLR itself were explored using the Mann–Whitney U-test and multivariable linear regression on log-transformed NLR, adjusting for age, sex, active cancer, CHF, CKD, anemia, and COVID-19 status.

Model performance was assessed using standard classification metrics, including sensitivity, specificity, positive predictive value (PPV), negative predictive value (NPV), accuracy, and Brier score, derived using standard formulas based on contingency tables. Discrimination was evaluated using the C-statistic derived from receiver-operating characteristic (ROC) analysis, with 95% CIs calculated by the DeLong method. ROC curves were compared using the DeLong test for correlated ROC curves, including the 95% CI for the difference in AUC. Incremental model performance with the addition of NLR was assessed by comparing nested logistic regression models using the likelihood ratio test (Δχ^2^) and change in Nagelkerke *R*^2^. Calibration of models with and without NLR was assessed using the Hosmer–Lemeshow goodness-of-fit test. Improvement in risk classification was evaluated using net reclassification improvement. Clinical utility was further assessed using decision curve analysis (DCA), which estimates net benefit across a range of threshold probabilities by comparing the association model with treat-all and treat-none strategies. Net benefit was evaluated across a range of threshold probabilities selected to span plausible decision points for escalating monitoring or level of care in acute PE, bracketing the cohort’s observed 30-day mortality rate rather than extreme values with limited practical application. Performance was further evaluated across ESC risk strata and clinically relevant subgroups, including cancer status, age, and sex.

Given the small sample size in the ESC high-risk group, analyses in this subgroup were considered exploratory due to limited statistical power. All analyses were performed using SPSS (IBM) and R version 4.6.0 software (R Foundation for Statistical Computing), with a 2-sided *P* value of <.05 considered statistically significant. The study was approved by the institutional review board with waiver of informed consent.

## 3 Results

### 3.1 Baseline characteristics

The study included 1525 patients with acute PE (mean age, 67.4 ± 17.2 years; 46% men). Patients were stratified by ESC risk category, with 707 (46.4%) classified as low risk, 789 (51.7%) as intermediate risk, and 29 (1.9%) as high risk. Active malignancy was present in 27.1% of patients, and 18.1% had CHF. Elevated NLR (≥7) was observed in 24.1% of the cohort; 30-day all-cause mortality occurred in 6.6% of patients (Table 1).

**Table 1:** Baseline characteristics of patients with acute pulmonary embolism.

Characteristic	Overall (N = 1525)
Demographics	
Age (y)	67.4 ± 17.2
Male sex	701 (46.0)
Female sex	824 (54.0)
ESC pulmonary embolism risk category	
Low risk	707 (46.4)
Intermediate risk	789 (51.7)
High risk	29 (1.9)
Comorbid conditions	
Active malignancy	413 (27.1)
Congestive heart failure	276 (18.1)
Chronic kidney disease	387 (25.4)
Chronic obstructive pulmonary disease	150 (9.8)
Anemia	498 (32.7)
Thrombocytopenia	204 (13.4)
Chemotherapy	193 (12.7)
Inflammatory marker	
Neutrophil-to-lymphocyte ratio ≥ 7	367 (24.1)
Outcomes	
30-d all-cause mortality	101 (6.6)

Values are shown as n (%) unless otherwise noted. Continuous variables are presented as mean ± SD. Thrombocytopenia was defined as an admission platelet count <150 × 10^9^/L. Race and ethnicity data were unavailable.

ESC = European Society of Cardiology.

### 3.2 Crude outcomes

At 30 days, mortality rates differed by NLR across ESC risk categories. In the low-risk group, mortality was 10.4% in patients with NLR ≥ 7 compared with 1.20% in those with NLR < 7 (absolute risk difference, +9.20%). In the intermediate-risk group, mortality was 16.2% vs 3.60% (absolute risk difference, +12.6%). In the high-risk group, mortality was similar between groups (83.3% vs 81.8%; absolute risk difference, +1.50%), although this is limited by the small sample size (Table 2).

**Table 2:** The 30-d Mortality by NLR category across ESC risk groups.

ESC risk group	NLR group	Deaths/total (%)	Absolute risk difference (%)
Low	<7	7/592 (1.20)	9.20
	≥7	12/115 (10.4)	
Intermediate	<7	20/555 (3.60)	12.6
	≥7	38/234 (16.2)	
High	<7	9/11 (81.8)	1.50
	≥7	15/18 (83.3)	

Mortality is presented as deaths/totals with percentages. Absolute risk differences represent the difference in mortality between patients with NLR of ≥7 and <7 within each ESC risk group.

ESC = European Society of Cardiology; NLR = neutrophil-to-lymphocyte ratio.

### 3.3 Unadjusted analysis

Elevated NLR (≥7) was associated with higher odds of 30-day mortality in ESC low- and intermediate-risk groups (Table 2).

### 3.4 Multivariable analysis

**Figure 1:**
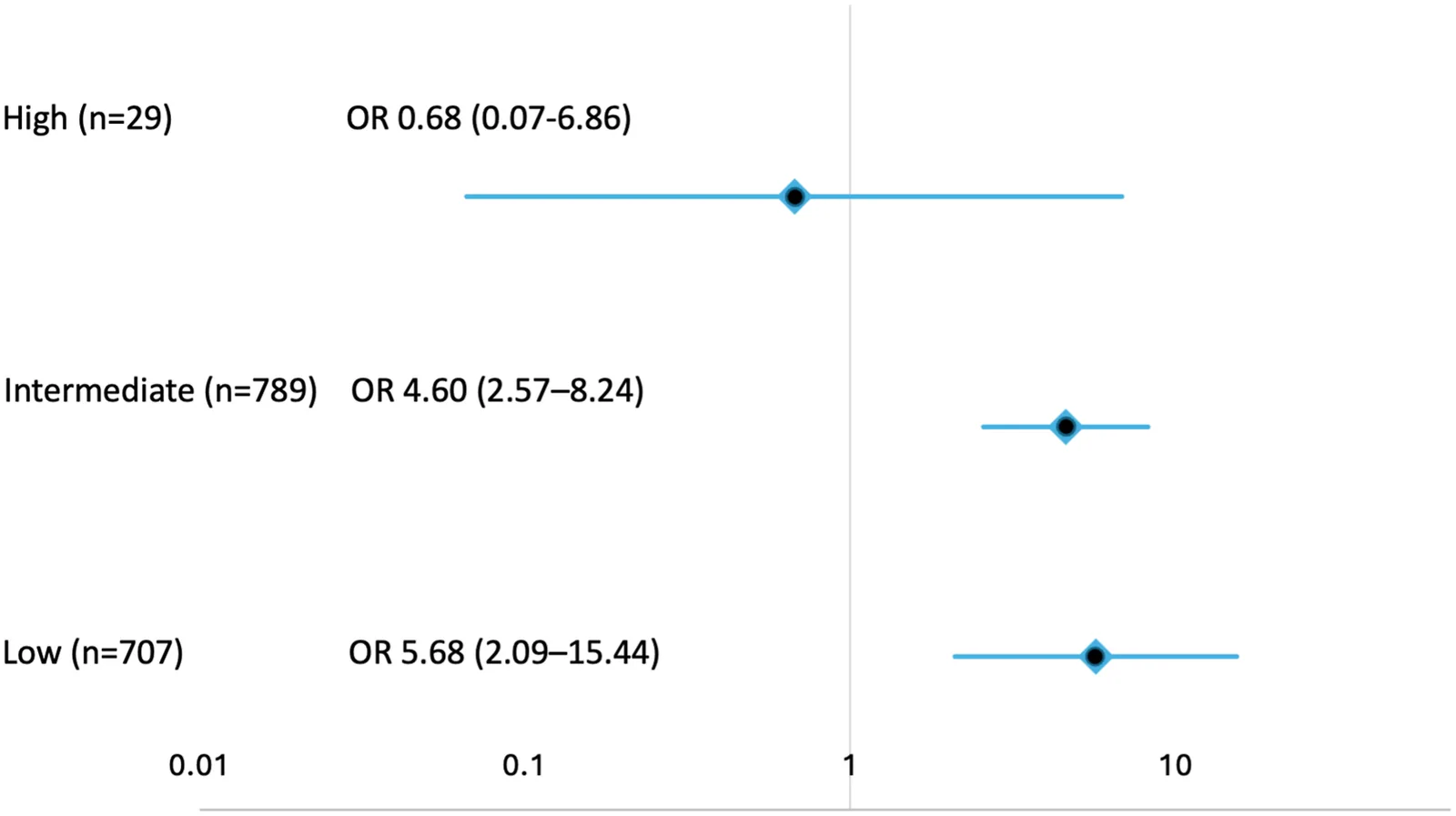
Elevated neutrophil-to-lymphocyte ratio (NLR) and 30-day mortality by European Society of Cardiology (ESC) risk. Forest plot showing adjusted odds ratios (ORs) and 95% CIs for the association between elevated NLR (NLR ≥ 7) and 30-day all-cause mortality across ESC pulmonary embolism risk categories. Models were adjusted for age, sex, active malignancy, and congestive heart failure. The vertical line denotes OR = 1.

After adjustment for age, sex, CHF, and active cancer, elevated NLR remained independently associated with 30-day mortality in both low- and intermediate-risk groups (Figure 1). In the low-risk group, patients with NLR ≥7 had 5.68-fold higher odds of death compared with those with NLR < 7 (OR, 5.68; 95% CI, 2.09-15.44). In the intermediate-risk group, NLR of ≥7 was associated with 4.60-fold higher odds of death (OR, 4.60; 95% CI, 2.57-8.24). These corresponded to absolute mortality differences of 9.2 and 12.6 percentage points in the low- and intermediate-risk groups, respectively (10.4% vs 1.2% and 16.2% vs 3.6%). Active cancer was also independently associated with mortality, while age, sex, and CHF were not significant predictors.

In the high-risk group, elevated NLR was not significantly associated with 30-day mortality after adjustment. Estimates were imprecise with wide CIs, and no covariates reached statistical significance, likely reflecting the sample size and reduced statistical power.

### 3.5 Sensitivity analyses

The association between elevated NLR and 30-day mortality was robust to sequential adjustment for CKD, COPD, troponin, BNP, and thrombocytopenia (adjusted ORs, 4.43-4.54 [Supplementary Table 1]; to Fine–Gray competing-risks regression accounting for non-PE causes of death, including in patients with active malignancy; and to formal testing of an NLR × cancer interaction, which was not significant [β = 0.42; *P* = .40]) (Supplementary Table 1). Modeling NLR as a continuous variable confirmed a dose–response relationship with mortality independent of the ≥7 cutoff (OR, 1.07 per 1-unit increase; *P* < .001), and active cancer and chemotherapy, rather than CHF, CKD, anemia, COVID-19 status, or thrombocytopenia, were the principal clinical correlates of NLR values themselves (Supplementary Tables 1-3, Supplementary Figure).

### 3.6 Model performance and incremental value

**Figure 2:**
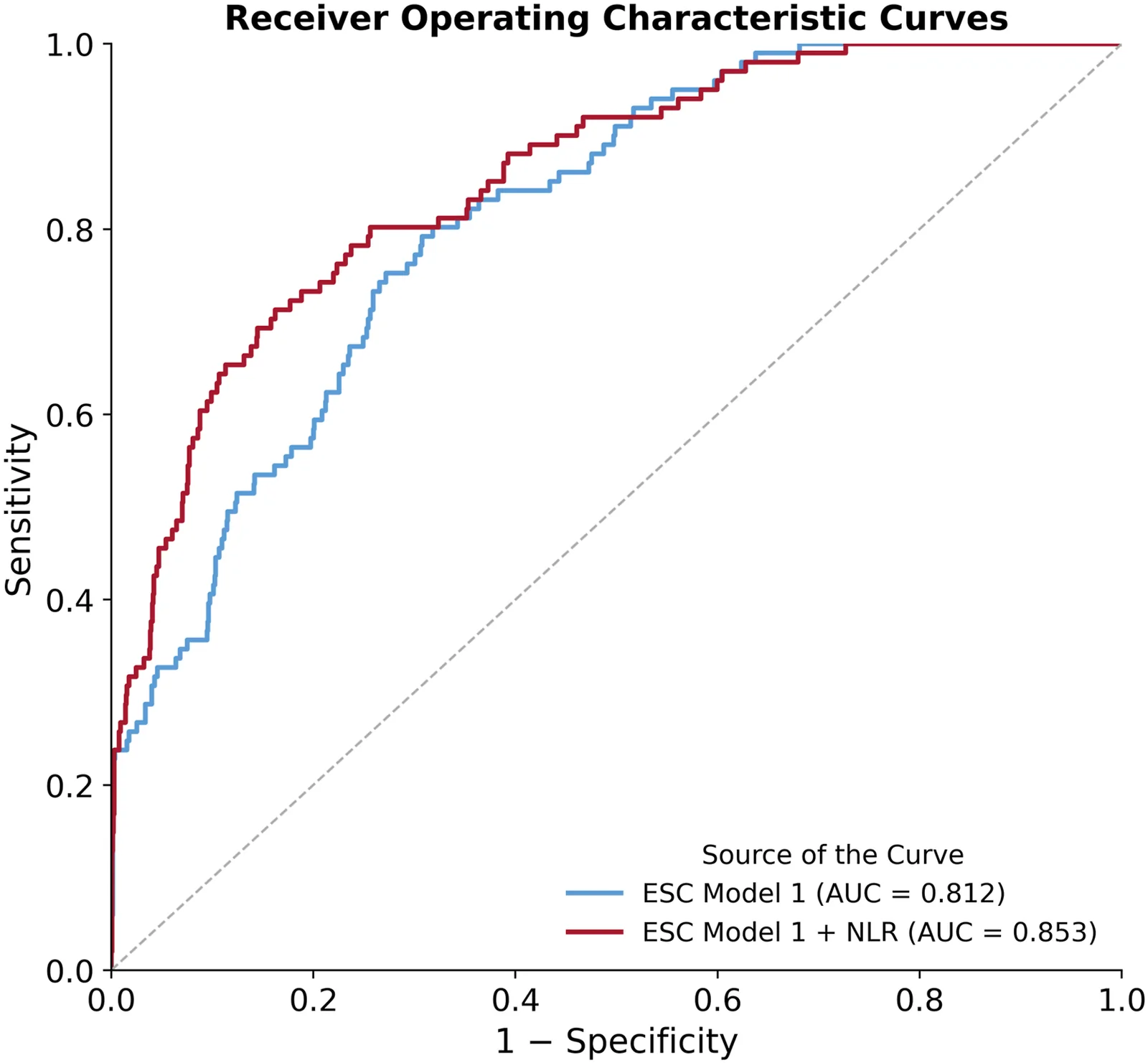
Receiver-operating characteristic (ROC) curves for association with 30-day mortality. ROC curves comparing a base model including European Society of Cardiology (ESC) risk category, age, sex, congestive heart failure, and active cancer (model 1) with an extended model incorporating neutrophil-to-lymphocyte ratio (NLR) (model 1 + NLR). Addition of NLR improved discrimination, with the C-statistic increasing from 0.812 to 0.853 (ΔAUC = 0.041; DeLong *P* = .003). AUC, area under the curve.

Incorporation of NLR into an ESC-based model was associated with improved overall model performance and discrimination for 30-day mortality. Addition of NLR resulted in a significant improvement in model fit (Δχ^2^ = 38.4; *P* < .001) and increased Nagelkerke *R*^2^ from 0.267 to 0.325. The C-statistic increased from 0.812 (95% CI, 0.774-0.850) to 0.853 (95% CI, 0.815-0.891; Δ = 0.041; 95% CI, 0.014-0.069; DeLong *P* = .003) (Figure 2), and net reclassification improvement was 0.19. The base model demonstrated evidence of miscalibration (Hosmer–Lemeshow χ^2^ = 23.7; df = 8; *P* = .003), whereas the NLR-enhanced model showed no statistically significant evidence of miscalibration (χ^2^ = 10.5; df = 8; *P* = .231), suggesting improved agreement between predicted and observed mortality rates (Table 3).

**Table 3:** Model performance of ESC-based risk model with and without NLR.

Metric	ESC model	ESC + NLR	Change	*P*
Panel A: model fit				
−2 log likelihood	577.8	539.4	−38.4	—
Δχ^2^ (df = 1)	—	38.4	—	<.001
Nagelkerke R^2^	0.267	0.325	+0.058	—
Panel B: discrimination and reclassification				
C-statistic	0.812	0.853	+0.041	.003
NRI	—	0.19	—	—

Logistic regression models for the 30-day mortality. The ESC model includes ESC risk category and clinical covariates (age, sex, congestive heart failure, and active cancer). The ESC + NLR model includes all variables in the ESC model with addition of NLR (≥7). Δχ^2^ represents the likelihood ratio test comparing nested models. Nagelkerke *R*^2^ reflects model fit. The C-statistic represents model discrimination. NRI quantifies improvement in risk classification. “—” indicates not applicable.

ESC = European Society of Cardiology; NLR = neutrophil-to-lymphocyte ratio; NRI = net reclassification improvement.

**Figure 3:**
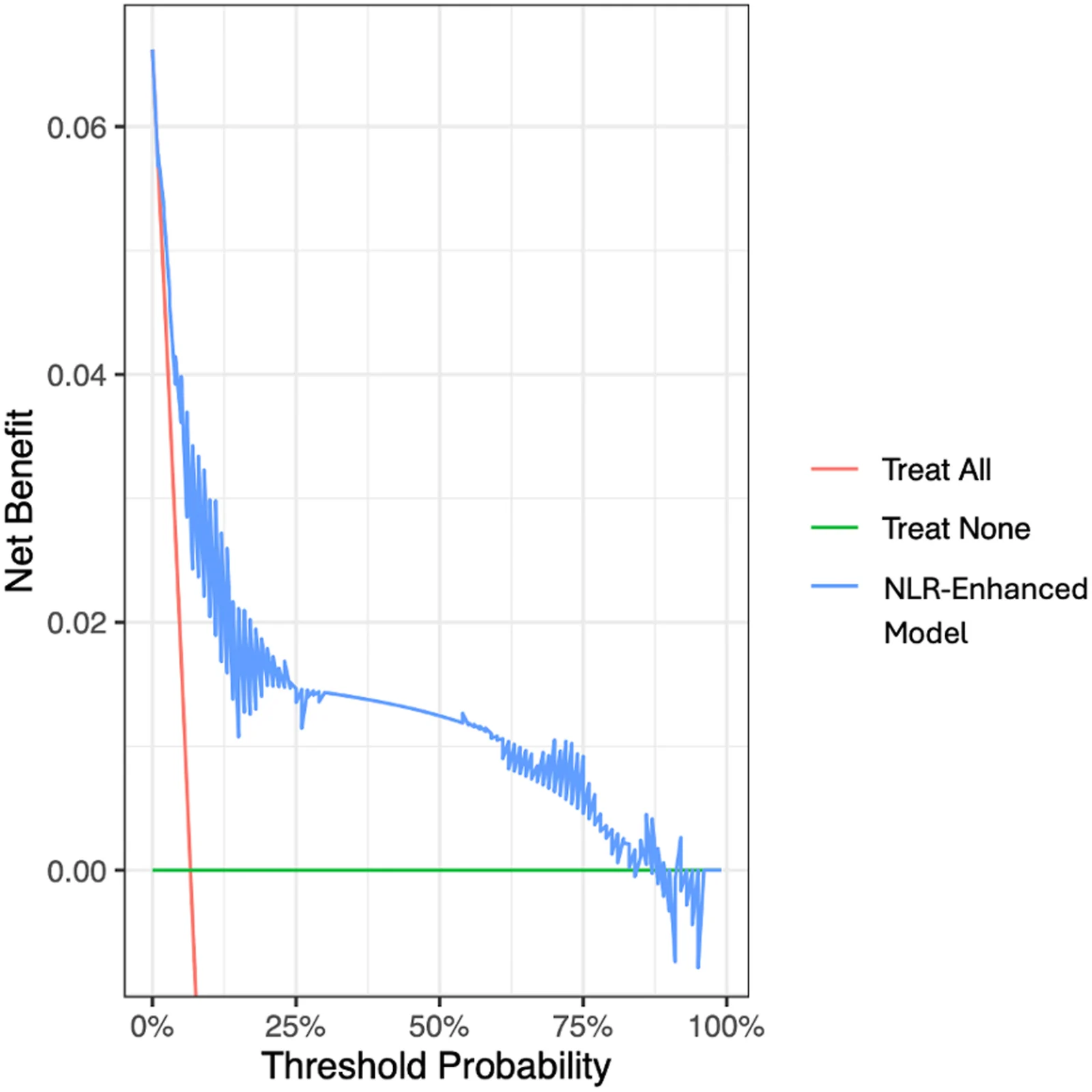
Decision curve analysis (DCA) of the neutrophil-to-lymphocyte ratio (NLR)-enhanced association model. DCA comparing the NLR-enhanced association model with treat-all and treat-none strategies for demonstrated prognostic value for 30-day mortality in acute pulmonary embolism. The NLR-enhanced model demonstrated greater net benefit across a broad range of clinically relevant threshold probabilities, particularly within lower to intermediate thresholds. Net benefit approaching zero at higher thresholds suggests diminishing clinical utility at extreme predicted risk probabilities.

DCA demonstrated greater net benefit for the NLR-enhanced model than treat-all or treat-none strategies across a clinically plausible range of threshold probabilities (Figure 3), where the treat-all strategy assumes intervention (eg, closer monitoring, escalation of care, or advanced therapy) regardless of predicted risk, and the treat-none strategy assumes no intervention. This indicates that using the model to guide selective escalation of monitoring or care would outperform either extreme approach, although it does not establish a specific NLR-based treatment protocol, and prospective validation of clinically actionable thresholds is needed.

### 3.7 Performance metrics

Elevated NLR (≥7) had a sensitivity of 64.4%, specificity of 78.8%, PPV of 17.7%, NPV of 96.8%, and overall accuracy of 77.8% for 30-day mortality (Brier score 0.22) (Supplementary Table 4). Performance remained broadly consistent across ESC risk strata (NPV, 98.8%, 96.4%, and 18.2% in low-, intermediate-, and high-risk groups, respectively) (Supplementary Table 5) and across cancer subgroups (sensitivity, 72.9% vs 52.4%; specificity, 68.1% vs 82.3% in patients with vs without active cancer).

## 4 Discussion

The principal clinical finding is that admission NLR identifies patients within ESC low- and intermediate-risk categories with markedly different 30-day mortality risks (10.4% vs 1.2% and 16.2% vs 3.6%), beyond what is captured by current ESC classification. In this retrospective cohort study of patients with acute PE, elevated NLR (NLR ≥ 7) was independently associated with increased 30-day mortality in ESC low- and intermediate-risk groups after multivariable adjustment for age, sex, CHF, and active cancer, suggesting that NLR provides clinically meaningful incremental prognostic information primarily in patients not already identified as high risk by ESC criteria. This study was designed to evaluate this prognostic association, not to estimate a causal effect of NLR on mortality. In plain terms, these findings do not establish that an elevated NLR causes death from PE; rather, it identifies a patient at higher overall risk.

NLR likely reflects systemic inflammation and physiologic stress in acute thromboembolic disease. As a nonspecific marker, NLR elevation may also reflect malignancy, occult infection, or other inflammatory processes rather than PE severity alone. The magnitude of this association is directionally consistent with, and somewhat greater than, that previously reported using the RIETE registry and Loyola University Medical Center cohort from which the NLR of ≥7 cutoff was derived (adjusted OR, 3.46; 95% CI, 2.60-4.60), providing an independent evaluation of this prognostic role rather than constituting a formal external validation study, and persisted after adjustment for CKD, COPD, troponin, BNP, and thrombocytopenia (Supplementary Table 1).

From a clinical standpoint, the most actionable signal from NLR is its NPV in ESC low- and intermediate-risk patients when NLR of <7 (98.8% and 96.4%, respectively) (Supplementary Tables 4 and 5): a low NLR may offer reassurance and support outpatient management, while its lower PPV (17.7%) means an elevated NLR should prompt closer monitoring rather than a specific treatment pathway. Incorporation of NLR into the ESC-based model also improved discrimination, calibration, and risk classification (see Results section), with DCA showing greater net benefit than treat-all or treat-none strategies across a broad range of threshold probabilities. Together, these findings support NLR’s use as an adjunctive tool layered onto existing ESC risk stratification, rather than a replacement for it or an independent guide to reperfusion, anticoagulation, or other treatment decisions.

In contrast, NLR did not meaningfully discriminate risk in the ESC high-risk group. This likely reflects the already elevated baseline risk in this population, driven by hemodynamic instability and markers of RV dysfunction that are predictive of poor prognosis. Additionally, the small sample size in the high-risk subgroup further limits interpretation and generalizability.

### 4.1 Limitations

Several limitations should be considered. First, this was a retrospective, single health system study, and residual confounding cannot be excluded. Second, NLR was assessed at a single time point and may not reflect dynamic changes in inflammatory status. Third, several potentially relevant variables were not systematically captured and could not be incorporated into the adjusted models, including anticoagulation strategies and adherence, social determinants of health, intensive care unit admission, hospital length of stay, use of advanced reperfusion therapies (systemic thrombolysis, catheter-directed thrombolysis or thrombectomy, and extracorporeal membrane oxygenation), and race and ethnicity. Fourth, internal or external validation was not performed, as this study was not designed to derive or validate a predictive model but rather to evaluate the prognostic association and performance of a single biomarker; accordingly, findings related to discrimination, reclassification, and DCA should be considered hypothesis generating. Fifth, other potentially relevant confounders—including coronary artery disease, active infection or sepsis, recent surgery, and other acute or chronic inflammatory conditions—were not systematically captured in this retrospective dataset; because NLR is a nonspecific inflammatory marker, unmeasured comorbid illness of this kind could still contribute in part to the observed association. Sixth, mortality ascertainment relied on death notifications documented within the electronic health record, and formal linkage with external state or national death registries was not performed; therefore, deaths occurring outside the health system that were not subsequently documented may have been missed. Seventh, granular clinical severity data were incompletely available: quantitative RV dysfunction variables were not retained, precluding reconstruction of intermediate-low and intermediate-high ESC subclassification, and data required to calculate the PESI or simplified PESI were not systematically available, precluding direct comparison of NLR’s prognostic performance against these established clinical risk scores. Finally, the small sample size in the high-risk group limited statistical power and precludes definitive conclusions in this subgroup.

## 5 Conclusion

Elevated NLR is independently associated with increased 30-day mortality in patients with acute PE classified as ESC low or intermediate risk. Incorporation of NLR into an ESC-based model improved model discrimination, risk classification, and net clinical benefit on DCA, supporting its potential clinical utility across selected threshold probabilities. However, given the retrospective design, these findings should be interpreted as hypothesis generating. The strongest clinical signal may be the consistently low short-term mortality observed among patients with NLR of <7 when interpreted alongside established PE risk-stratification frameworks. Prospective studies are warranted to determine whether incorporation of NLR into clinical decision making improves patient outcomes.
