# Supplementary material for: Neutrophil-to-lymphocyte ratio is associated with 30-day mortality in acute pulmonary embolism across European Society of Cardiology risk categories
Source: Res Pract Thromb Haemost. 2026 Aug 21;10(6):106918. doi: 10.1016/j.rpth.2026.106918 (PMC13579932; doi:10.1016/j.rpth.2026.106918)
Supplement: Supplementary file 1 [file mmc1.docx]

**Supplementary Material**

***Manuscript: "Neutrophil-to-Lymphocyte Ratio Is Associated With 30-Day Mortality in Acute Pulmonary Embolism Across European Society of Cardiology Risk Categories"***

**Supplementary Table S1.** Sensitivity Analyses for the Association Between Elevated NLR (≥7) and 30-Day Mortality

| **Sensitivity Analysis** | **Additional Adjustment Variables** | **Adjusted OR (95% CI) for NLR ≥7** | **p-value** |
| --- | --- | --- | --- |
| Base multivariable model | Age, sex, active cancer, CHF, ESC risk category | 4.50 (2.79–7.38) | <0.001 |
| + Chronic kidney disease, COPD | Base + CKD, COPD | 4.53 (2.80–7.43) | <0.001 |
| + Troponin, BNP status | Above + troponin, BNP status | 4.43 (2.73–7.29) | <0.001 |
| + Thrombocytopenia (platelet <150 ×10⁹/L) | Age, active cancer, CHF, CKD, ESC risk category, thrombocytopenia | 4.54 (2.80–7.38) | <0.001 |
| Continuous NLR (per 1-unit increase) | ESC risk category | 1.07 (β = 0.067) | <0.001 |
| NLR × cancer interaction term | ESC risk category, age, sex, active cancer, CHF | β = 0.42 | 0.40 |
| Fine-Gray: PE-specific death (full cohort, SHR for NLR ≥7) | Age, cancer, CHF, CKD, COPD, ESC risk category | 3.19 (1.61–6.34) | <0.001 |
| Fine-Gray: non-PE competing death (full cohort, SHR for NLR ≥7) | Age, cancer, CHF, CKD, COPD, ESC risk category | 3.65 (2.06–6.49) | <0.001 |
| Fine-Gray: PE-specific death (cancer subgroup, SHR for NLR ≥7) | Age, ESC risk category | 3.06 (1.02–9.13) | 0.045 |
| Fine-Gray: non-PE competing death (cancer subgroup, SHR for NLR ≥7) | Age, ESC risk category | 4.18 (2.02–8.66) | <0.001 |

The base multivariable model was sequentially expanded to include additional comorbidities and biomarkers as noted. The thrombocytopenia sensitivity analysis was fit as a separate model adjusting for the variables listed rather than cumulatively with the CKD/COPD and troponin/BNP models. Fine-Gray competing risks analyses report subdistribution hazard ratios (SHR), not odds ratios, and treat PE-specific and non-PE death as competing events; PE-specific death was classified per the RIETE Fatal PE definition. CHF = congestive heart failure; CKD = chronic kidney disease; COPD = chronic obstructive pulmonary disease; BNP = brain natriuretic peptide; ESC = European Society of Cardiology; SHR = subdistribution hazard ratio.

**Supplementary Table S2.** NLR Distribution by Cardiac Biomarker and Lactate Status

| **Biomarker** | **n Positive / n Negative** | **Median NLR (Positive)** | **Median NLR (Negative)** | **p-value** |
| --- | --- | --- | --- | --- |
| Troponin | 722 / 803 | 4.70 | 3.31 | <0.001 |
| BNP | 526 / 999 | 5.04 | 3.30 | <0.001 |
| Lactate* | 188 / 383 | 5.27 | 4.55 | 0.197 |

Median admission NLR compared by Mann-Whitney U test across troponin, BNP, and lactate positivity. Troponin and BNP status were available for all patients in the analytic cohort. *Lactate was drawn in only 571 of 1,525 patients (37.4%) and was not part of ESC risk classification; this comparison should be interpreted with caution given substantial missingness. NLR = neutrophil-to-lymphocyte ratio; BNP = brain natriuretic peptide.

**Supplementary Table S3.** NLR by Comorbidity and Treatment Status

| **Variable** | **n (Yes/No)** | **Median NLR (Yes)** | **Median NLR (No)** | **p-value** |
| --- | --- | --- | --- | --- |
| Active cancer | 413 / 1,112 | 5.15 | 3.56 | <0.001 |
| Chemotherapy | 193 / 1,332 | 5.33 | 3.80 | <0.001 |
| CHF | 276 / 1,249 | 4.11 | 3.86 | 0.776 |
| CKD | 387 / 1,138 | 4.27 | 3.81 | 0.055 |
| Anemia | 498 / 1,027 | 4.03 | 3.85 | 0.120 |
| COVID-19 status | 336 / 1,189 | 3.88 | 3.91 | 0.943 |
| Thrombocytopenia (platelet <150 ×10⁹/L) | 204 / 1,321 | 3.92 | 3.90 | 0.920 |

Median admission NLR compared by Mann-Whitney U test across each variable. Active cancer and chemotherapy were associated with significantly higher NLR values; CHF, CKD, anemia, COVID-19 status, and thrombocytopenia were not. CHF = congestive heart failure; CKD = chronic kidney disease; NLR = neutrophil-to-lymphocyte ratio.

**Supplementary Table S4. Overall Performance of NLR≥7 Associated with 30-Day Mortality**

| **Metric** | **Value** |
| --- | --- |
| Sensitivity | 64.4% |
| Specificity | 78.8% |
| Positive Predictive Value | 17.7% |
| Negative Predictive Value | 96.8% |
| Accuracy | 77.8% |
| Brier score | 0.22 |

Performance measures are shown for NLR≥7 as a binary predictor for 30-day mortality.

**Supplementary Table S5.** Performance of Elevated NLR (≥7) for 30-Day Mortality, Stratified by ESC Risk Category

| **ESC Risk Category** | **Sensitivity** | **Specificity** | **Negative Predictive Value** |
| --- | --- | --- | --- |
| Low risk | 63.2% | 85.0% | 98.8% |
| Intermediate risk | 65.5% | 73.2% | 96.4% |
| High risk | 62.5% | 40.0% | 18.2% |

Sensitivity, specificity, and negative predictive value of elevated NLR (≥7) for 30-day all-cause mortality within each ESC risk category. ESC = European Society of Cardiology.

**Supplementary Figure S1.** Distribution of NLR and Its Component Blood Count Parameters by NLR Group

Box-and-whisker plots (log scale) of NLR and its two component blood counts, stratified by NLR group (<7 vs ≥7). NLR: median 3.00 (IQR 1.74–4.52) in the NLR<7 group vs. 10.67 (IQR 8.50–14.73) in the NLR≥7 group. Absolute neutrophil count (ANC, ×10⁹/L): median 6.10 (IQR 4.40–7.80) vs. 10.00 (IQR 7.75–13.00). Absolute lymphocyte count (ALC, ×10⁹/L): median 2.00 (IQR 1.40–2.90) vs. 0.85 (IQR 0.60–1.10). All between-group differences were significant (Mann-Whitney U, p < 0.001). Elevated NLR reflects both a higher neutrophil count and a lower lymphocyte count relative to the NLR<7 group. Boxes show the median and interquartile range (IQR); whiskers extend to 1.5× IQR; points beyond the whiskers are shown individually.
